# Usefulness of silent magnetic resonance angiography (MRA) for the diagnosis of atherosclerosis of the internal carotid artery siphon in comparison with time-of-flight MRA

**DOI:** 10.1186/s40001-022-00673-4

**Published:** 2022-03-21

**Authors:** Rui Li, Song Jin, Tao Wu, Xiao Zang, Meng Li, Jinfeng Li

**Affiliations:** 1 Department of Medical Imaging, Tianjin Huanhu Hospital, 6 Jizhao Road, Tianjin, 300350 China; 2 GE Healthcare MR Enhanced Application Team, 1 Tongji South Road, Beijing, 100176 China; 3 Department of Radiology, The First Medical Center of PLA General Hospital, 28 Fuxing Road, Beijing, 100853 China; 4 Department of Medical Imaging, Affiliated Huanhu Hospital of Nankai University, 6 Jizhao Road, Tianjin, 300350 China

**Keywords:** Magnetic resonance angiography, Silent MRA, TOF MRA, Digital subtraction angiography, Atherosclerosis, Internal carotid artery siphon, Diagnostic efficacy

## Abstract

**Background and purpose:**

Flow visualization in 3D time-of-flight MRA (3D-TOF MRA) may be limited for internal carotid artery siphon owing to turbulent artifact. The purpose of this study was to compare the usefulness of Silent MRA and 3D-TOF MRA to assess atherosclerosis of the internal carotid artery siphon.

**Material and methods:**

A total of 106 patients with suspected cerebrovascular disease were included. All patients were scanned with Silent MRA and 3D-TOF MRA sequences and also underwent DSA examination. Two observers independently assessed the TOF MRA and Silent MRA images of atherosclerosis of the internal carotid artery siphon. The diagnostic efficacy of two MRA methods in evaluating atherosclerosis of the carotid siphon was performed by using receiver operating characteristic (ROC) curve analysis. Interobserver reliability was also assessed using weighted kappa statistics.

**Results:**

Image of Silent MRA sequence had higher subjective evaluation scores and significantly high CNR between the carotid siphon and the background tissues than the image of 3D-TOF MRA sequence (*P* < 0.05). The AUC was 0.928 (95% CI 0.909–0.986) for Silent MRA, which was significantly higher than that of 3D-TOF MRA (0.671, 95% CI 0.610–0.801, *P* < 0.05). Silent MRA had high sensitivity, specificity and accuracy than 3D-TOF MRA for visualization of the carotid siphon.

**Conclusions:**

Silent MRA as a new angiographic modality is superior to 3D-TOF MRA for visualization of the carotid siphon, and maybe an alternative to 3D-TOF MRA in the diagnosis of atherosclerosis of the carotid siphon.

## Introduction

Intracranial atherosclerosis (IAS), leading to cerebrovascular stenosis and/or occlusion, could be a major cause for acute cerebral accidents [1], which is a degenerative disease of the arteries that results in the formation of plaques made of necrotic cells, fatty streaks, calcification, and cholesterol crystals [2, 3]. Atherosclerotic disease often involves the intracranial arteries, including internal carotid arteries (ICA), intracranial vertebral arteries (IVA), basilar arteries (BA), middle cerebral arteries (MCA), most predominantly in the internal carotid artery, especially in the internal carotid siphon because of its tortuous anatomical configuration [4, 5].

Multiple imaging techniques, such as transcranial Doppler ultrasonography (TCD), CT angiography (CTA) and MR angiography (MRA), are now routinely available for detection and diagnosis of atherosclerosis in clinical practice [6–8]. Although digital subtraction angiography (DSA) technique has been widely recognized as the gold standard for the diagnosis of cerebrovascular diseases, its application in preoperative screening of cerebrovascular diseases was limited by its disadvantages, such as invasiveness and contrast-related complications, and increased radiation dose exposure [1, 9]. In addition, for patients with intracranial atherosclerotic disease, these noninvasive techniques, as mentioned above, have been validated against clinical outcomes and events, which were frequently preferred during the screening, diagnosis and follow-up [7, 10, 11].

Three dimensional time-of-flight MRA (3D-TOF MRA) has been widely used as a method to evaluate cerebrovascular status due to its advantages, such as high resolution, free of contrast and radiation, and simple operation [12, 13]. 3D-TOF MRA mainly exploits the inflow-enhancement effect and depends on blood flow velocity. However, it is greatly influenced by hemodynamics and limited in visualizing the fine structures of intracranial and external arteries, especially in areas with complex vessel direction and hemodynamics, such as the internal carotid artery siphon [14]. The zero echo time (ZTE)-MRA, Silent MRA, is a novel technique developed in recent years, which is based on arterial spin labeling (using water molecules within the arterial blood as an endogenous tracer) and is not influenced by the blood flow state, and can evaluate whether the blood vessel is narrowed and the degree of stenosis more realistically [15, 16].

The purpose of this study was to compare Silent MRA with 3D-TOF MRA for the evaluation of atherosclerosis of the internal carotid artery siphon, and compare their diagnostic efficacy by using DSA as a gold standard.

## Material and methods

### Study population

A total of 106 patients with cerebrovascular disease who underwent DSA examination in our hospital from June 2018 to January 2020 were retrospectively included. There were 49 male and 57 female with an average age of 65.9 years (range 47 to 94 years). The study was approved by the Ethical Committee of our hospital, and written informed consent was obtained from each subject before the study. Patients who could not tolerate MRI were excluded.

### MRI protocol

Magnetic resonance images were acquired using a 3.0 Tesla MR scanner (Discovery 750w 3.0T, GE Healthcare, Milwaukee, USA) with a 32-channel head coil (GE Healthcare). Vacuum cushions were applied to reduce the noise and movement artifact. The 3D-TOF MRA sequence was scanned in the oblique plane with the following parameters: TR = 23 ms, TE = 2.8 ms, FOV = 240 × 211 mm^2^, slice thickness = 1.4 mm, Matrix = 384 × 256, NEX = 0.85. The Silent MRA sequence was acquired in sagittal plane with the following parameters: TR = 826 ms, TE = 0 ms, FOV = 200 × 200 mm^2^, slice thickness = 1.2 mm, Matrix = 166 × 166 mm, NEX = 1. The technical details for all the MRI sequences are presented in Table 1.

**Table 1:** Scan parameters of 3D-TOF MRA and Silent MRA

Parameters	3D-TOF MRA	Silent MRA
Scan plane	Oblique plane	Sagittal plane
Acquisition type	3-dimensional	3-dimensional
TR/TE (ms)	23/2.8	826/0
FOV (mm^2^)	240 × 211	200 × 200
Matrix	384 × 256	166 × 166
Bandwidth (Hz)	31.20	31.20
Flip angle (°)	20	3
Slice thickness (mm)	1.4	1.2
NEX	0.85	1
Acquisition time (min:s)	4:16	6:17

*TE* echo time, *TR* repetition time, *FOV* field of view, *NEX* number of excitations, *TOF* time of flight, *MRA* magnetic resonance angiography

### Image assessment

The original images of the Silent MRA and 3D-TOF MRA sequences were transmitted to an AW4.6 workstation (GE Healthcare) for maximum intensity projection (MIP). Then the raw data and MIP images were analyzed by subjective and objective image quality evaluation, respectively. Subjective image quality assessment was performed blinded by two neuroradiologists with 10 years of experience using a 5-point scale to evaluate image uniformity, presence/absence of artifacts, the visualization of the carotid siphon, and overall image quality.

**Fig. 1:**
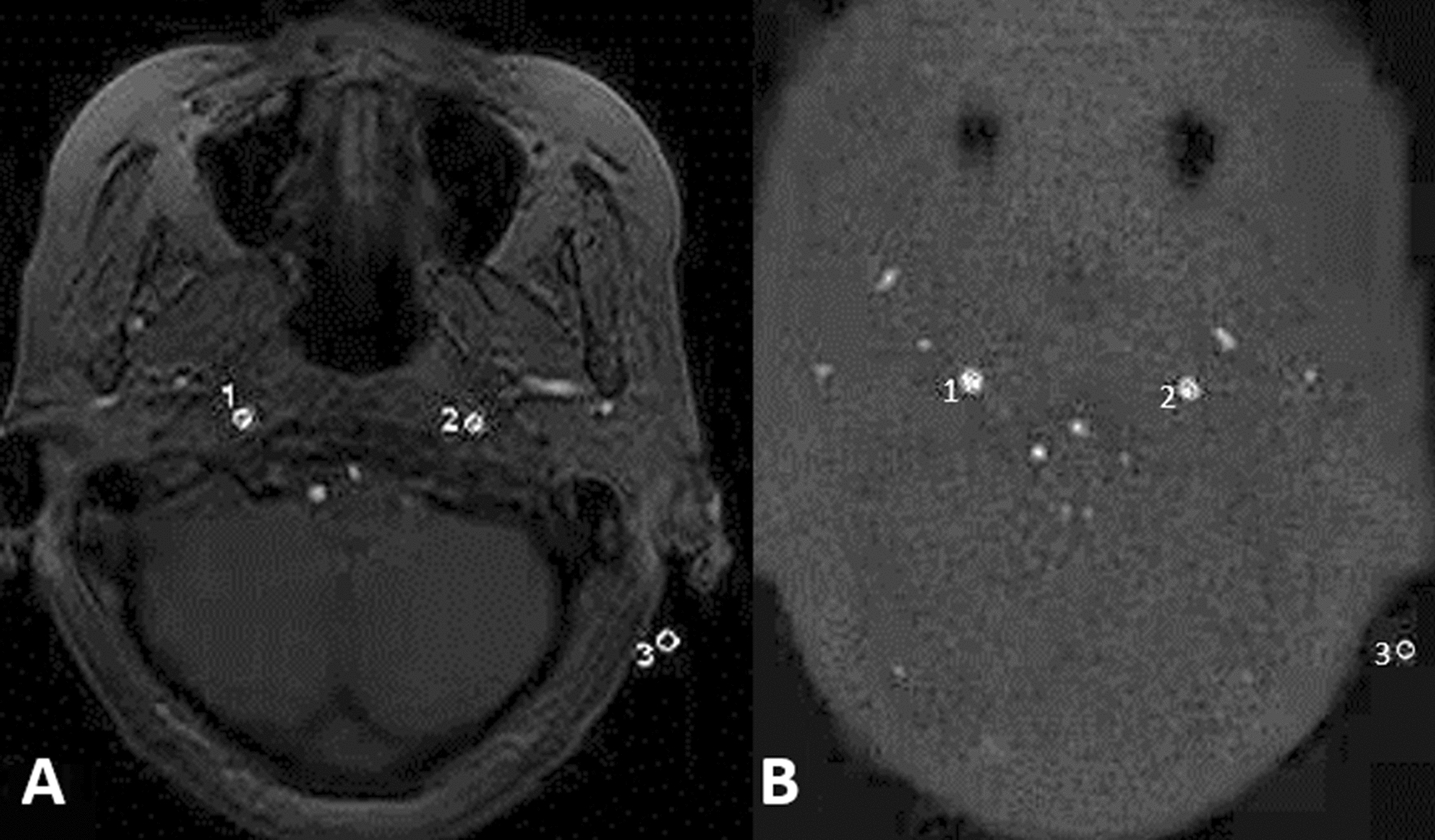
Measurement of SNR and CNR of internal carotid artery by 3D-TOF MRA (**A**) and Silent MRA (**B**)

**Fig. 2:**
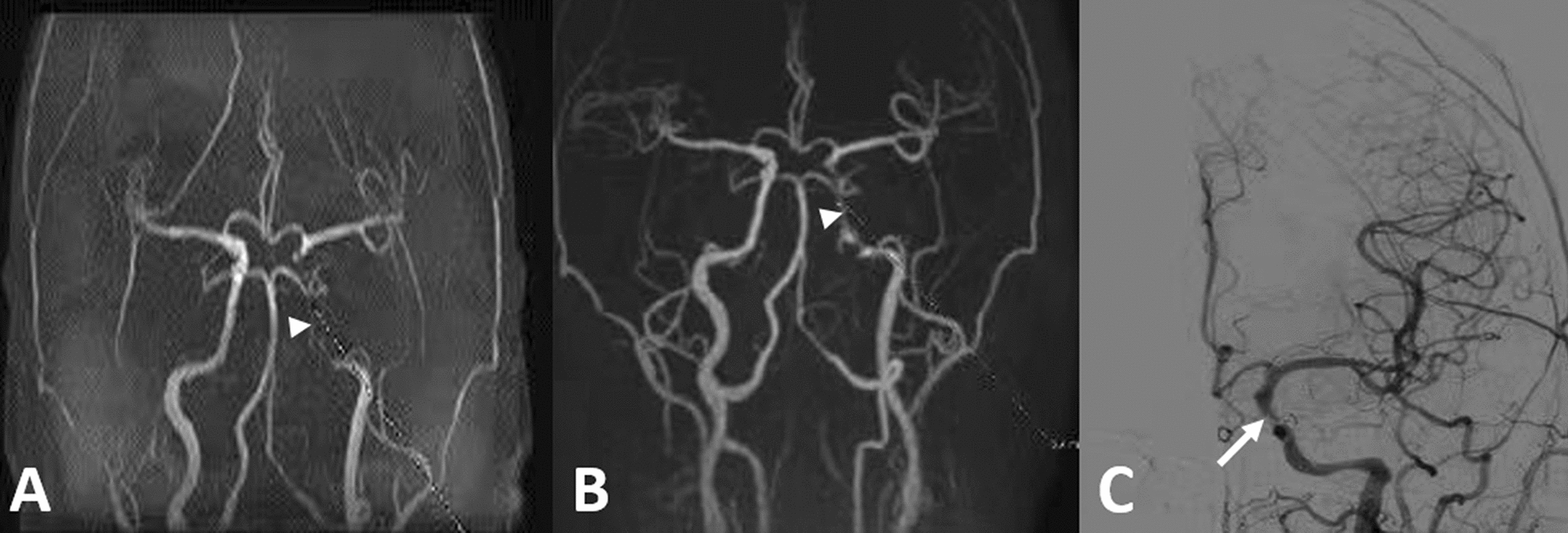
**A** 3D-TOF MRA shows almost lost the signal, almost occlusion, in the siphon segment of left internal carotid artery (white arrowhead). **B** Silent MRA shows focal stenosis in the same as **A** (white arrowhead). **C** DSA also demonstrates focal stenosis in the siphon segment of left internal carotid artery (white arrow), in consistent with Silent MRA

The quantitative measurement included signal-to-noise ratio (SNR) for the carotid siphon, and contrast-to-noise ratio (CNR) of the carotid siphon with respect to the background. The region of interest (ROIs) were delineated in the carotid siphon, background tissues and the corresponding background area on the original images of the Silent MRA and 3D-TOF MRA sequences. The SNR for the carotid siphon, and CNR of the carotid siphon with respect to the background were calculated according to the following formula: SNR = SI/SDnoise, CNR = (SNR1 − SNR2)/SDnoise, where SI1 and SI2 represent the signal intensity (SI) at the carotid siphon and the SI of the corresponding background tissues, respectively, SD noise represents the standard deviation of the signal intensity of background noise on corresponding images. The results obtained were used for further statistical analysis (Fig. 1). DSA was used as the gold standard, the consistency between the Silent MRA and DSA images in the visualization of the carotid siphon were analyzed to determine the efficacy of Silent MRA in diagnosing of atherosclerosis of the carotid siphon (Fig. 2).

### Statistical analysis

All statistical analyses were performed using SPSS 17.0 software (IBM SPSS Inc., Chicago, IL, USA). Result of subjective image quality assessment was analyzed using the Kruskal–Wallis rank sum test. A weighted kappa (*κ*) test was used to analyze interobserver agreement among the two neuroradiologists. A weighted *κ* value was interpreted as follows: excellent (> 0.81), good (0.61–0.81), moderate (0.41–0.60), poor (< 0.40). Result of objective image quality assessment was analyzed using the two-sample *t*-test. In addition, the diagnostic efficacy of Silent MRA and 3D-TOF MRA in evaluating the degree of atherosclerotic stenosis in the internal carotid artery siphon was assessed by using DSA as the gold standard. The receiver operating characteristic (ROC) curves of Silent MRA and 3D-TOF MRA for diagnosis of atherosclerosis of the carotid siphon were drawn. Area under ROC curves (AUCs) and their 95% confidence intervals (CIs) were calculated. A *P* value less than 0.05 was considered statistically significant.

## Results

### Subjective image quality assessment

Image of Silent MRA sequence had higher subjective assessment scores (for uniformity, artifact, visualization of the carotid siphon and overall image quality) than the image of 3D-TOF MRA sequence (both *P* < 0.05, Table 2). The weighted *κ* value of image score evaluated by two experienced radiologists was 0.88, which indicated good consistency.

**Table 2:** Comparison of subjective image quality assessment scores between images of Silent MRA and 3D-TOF MRA sequences (mean ± SD)

Sequences	Uniformity	Artifact	Visualization of the internal carotid artery siphon	Overall image quality
Silent MRA	4.92 ± 0.82	4.56 ± 1.09	4.92 ± 0.76	4.79 ± 0.91
3D-TOF MRA	2.39 ± 0.56	2.89 ± 0.90	2.81 ± 0.87	3.07 ± 0.86
*Z*	32.65	24.21	31.08	25.69
*P*	< 0.05	< 0.05	< 0.05	< 0.05

### Objective image quality assessment

Image of Silent MRA sequence showed significantly high CNR between the carotid siphon and the background tissues than the image of 3D-TOF MRA sequence (*P* < 0.05) (Table 3). There was no significant difference in the SNR for the carotid siphon between the two sequences (*P* > 0.05, Table 3).

**Table 3:** Comparison of results of objective image assessment between images of Silent MRA and 3D-TOF MRA sequences (mean ± SD)

Sequences	SNR	CNR
Silent MRA	163.38 ± 7.02	92.98 ± 8.23
3D-TOF MRA	166.19 ± 10.32	70.01 ± 9.08
*F*	1.89	19.76
*P*	> 0.05	< 0.05

### The difference in AUC between silent MRA and 3D-TOF MRA for the diagnosis of atherosclerosis of the carotid siphon

**Fig. 3:**
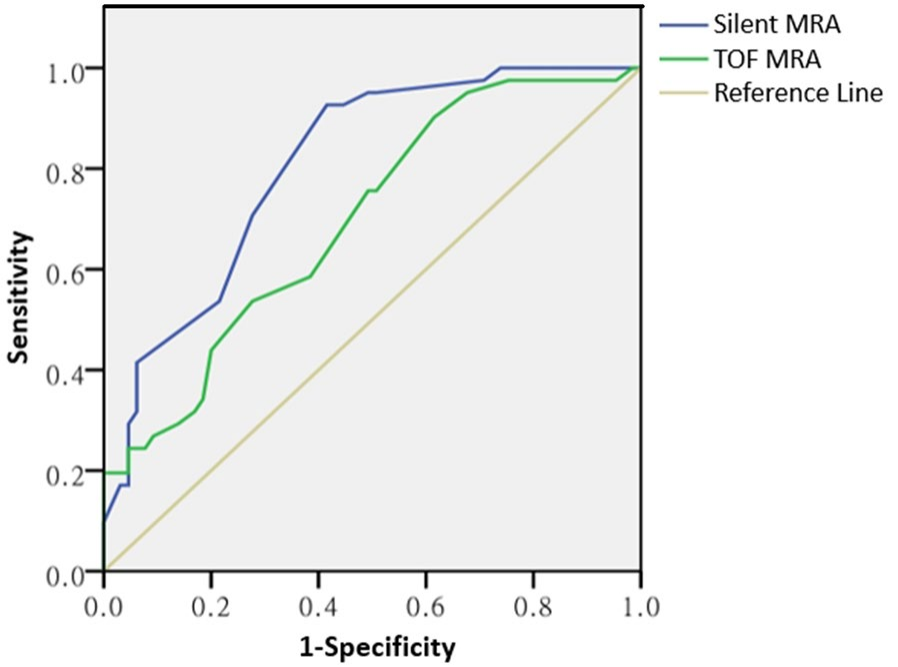
ROC curves for diagnosis of carotid siphon atherosclerosis in Silent MRA (blue line) and 3D-TOF MRA (green line). The AUC of Silent MRA was 0.928, significantly higher than that of the 3D-TOF MRA (AUC = 0.671, *P* < 0.05). *MRA* magnetic resonance angiography, *TOF* time-of-flight

Among106 patients with suspected atherosclerosis of the carotid siphon, 98 patients were confirmed by DSA. Of those 98 patients, 95 patients were detected, and 3 patients were not detected by Silent MRA, and Silent MRA was positive in one DSA-negative patient. Of those 98 patients confirmed by DSA, 79 patients were detected and 19 patients were not detected by 3D-TOF MRA. Moreover, 3D-TOF MRA was positive in 7 DSA-negative patients. The AUC was 0.928 (95% CI 0.909–0.986) for Silent MRA, and 0.671 (95% CI 0.610–0.801) for 3D-TOF MRA. There were statistically significant differences in the AUC between Silent MRA and 3D-TOF MRA (*P* < 0.05, Fig. 3).

## Discussion

In our present study, the usefulness of Silent MRA and 3D-TOF MRA for the evaluation atherosclerosis of the internal carotid artery siphon were compared. Our principal findings can be summarized as follows: (i) Silent MRA was feasible to depict cerebrovascular conditions with dramatically reduced acoustic noise, higher signal homogeneity, and higher quality of venous signal suppression. (ii) Silent MRA was superior to 3D-TOF MRA for visualization of the carotid siphon, despite the relatively small sample size.

Magnetic resonance imaging (MRA) using 3D-TOF MRA technique, in clinical practice, has been modality most widely used to evaluate cerebral vessels [8, 17]. It has the advantages of contrast-free, invasiveness, and no radiation exposure, but it still has some shortcomings. Areas with slow blood flow or small blood vessels at the distal end may be saturated and show low signal and areas with complex hemodynamics, such as the carotid siphon, can also accelerate the loss of phase coherence of protons, and cause signal attenuation, leading to false-positive results or exaggeration of the extent of the disease, so it is more difficult to evaluate with 3D-TOF MRA.

The Silent MRA, a novel MRI angiographic modality, integrates both continuous arterial spin labeling (ASL) and ZTE radial acquisition readout, which minimize the sensitivity to magnetic field inhomogeneity and complex blood flow [9, 18], which uses water molecules within the arterial blood as an endogenous tracer. A post-labeling delay of 2 s is necessary to allow the labeled arterial blood reach the imaging region, and reduce the signal-to-noise ratio [16]. Therefore, Silent MRA is less sensitive to hemodynamics and provides good visualization compared with 3D-TOF MRA. In addition, Silent MRA uses minimal readout gradient switching, which can not only achieve quiet scanning, but also reduce the eddy current effect and a series of artifacts caused by the eddy current effects, and ensure the accurate filling of the K space [19]. Irie et al. [20] and Ryu et al. [11] used 3D-TOF MRA and Silent MRA to evaluate intracranial aneurysms treated with stent-assisted coil embolization, and results showed that Silent MRA was superior than that of 3D-TOF MRA in evaluation of intracranial aneurysms after stent-assisted coil embolization. Tomura et al. [21] reported silent MRA visualized feeders and drainers in arteriovenous malformations (AVMs) significantly better than did TOF MRA.

Silent MRA is acquired using a 3D acquisition, the readout gradient precedes the radio frequency excitation, and slice selection gradient cannot be applied, so 2D acquisition cannot be performed. The disadvantages of Silent MRA include the long scanning time (large coverage), and high resolution (1.2 mm thickness)-induced blurring of vessel edges [22]. Although Silent MRA has limited spatial resolution and requires additional acquisition time, the MRI method may have the potential to improve the imaging quality of carotid artery [23].

In the present study, we investigated the usefulness of Silent MRA for the visualization of the carotid siphon, a tortuous vessel segment with complex hemodynamics, our results showed that Silent MRA provided excellent image quality of the carotid siphon, and showed a higher consistency with DSA. Silent MRA appears to better visualize the carotid siphon than 3D-TOF MRA. Silent MRA eliminates the influence of blood flow or hemodynamics, and more realistically and accurately reflects the blood flow of the carotid siphon without false positive results and exaggeration of stenosis. However, the limitation of the study is the small sample size, larger studies are required for further verifying superiority to Silent MRA.

## Conclusions

Silent MRA is a new angiographic modality, which is superior to 3D-TOF MRA for visualization of the carotid siphon segment, and may possibly replace the 3D-TOF MRA in the diagnosis of atherosclerosis of the carotid siphon. Although this novel image sequence is only currently available to a limited commercial 3.0 Tesla scanner, it is likely that this potential MRA method could be widely applied in the future after further development.

## Data Availability

The datasets collected and analyzed during the current study are available from the corresponding author upon reasonable request.

## References

[1] Chaoqun G (2018). Analysis of radiation effects in digital subtraction angiography of intracranial artery stenosis. J World Neurosurg.

[2] Qureshi AI, Caplan LR (2014). Intracranial atherosclerosis. Lancet.

[3] Falk E (2006). Pathogenesis of atherosclerosis. J Am Coll Cardiol.

[4] Wong LK (2006). Global burden of intracranial atherosclerosis. Int J Stroke.

[5] Haussen DC, Gaynor BG, Johnson JN, Peterson EC, Elhammady MS, Aziz-Sultan MA, Yavagal DR (2014). Carotid siphon calcification impact on revascularization and outcome in stroke intervention. Clin Neurol Neurosurg.

[6] Sharma VK, Wong KS, Alexandrov AV (2016). Transcranial Doppler. Front Neurol Neurosci.

[7] Byrne D, Walsh JP, Sugrue G, Stanley E, Marnane M, Walsh CD, Kelly P, Murphy S, Kavanagh EC, MacMahon PJ (2018). Subtraction multiphase CT angiography: a new technique for faster detection of intracranial arterial occlusions. Eur Radiol.

[8] Sarikaya B, Colip C, Hwang WD, Hippe DS, Zhu C, Sun J, Balu N, Yuan C, Mossa-Basha M (2021). Comparison of time-of-flight MR angiography and intracranial vessel wall MRI for luminal measurements relative to CT angiography. Br J Radiol.

[9] Shang S, Ye J, Luo X, Qu J, Zhen Y, Wu J (2017). Follow-up assessment of coiled intracranial aneurysms using zTE MRA as compared with TOF MRA: a preliminary image quality study. Eur Radiol.

[10] Ozsarlak O, Van Goethem JW, Maes M, Parizel PM (2004). MR angiography of the intracranial vessels: technical aspects and clinical applications. Neuroradiology.

[11] Ryu KH, Baek HJ, Moon JI, Choi BH, Park SE, Ha JY, Park H, Kim SS, Kim JS, Cho SB, Carl M (2020). Usefulness of noncontrast-enhanced silent magnetic resonance angiography (MRA) for treated intracranial aneurysm follow-up in comparison with time-of-flight MRA. Neurosurgery.

[12] Gamaleldin OA, Donia MM, Elsebaie NA, Abdelkhalek Abdelrazek A, Rayan T, Khalifa MH (2020). Role of fused three-dimensional time-of-flight magnetic resonance angiography and 3-dimensional T2-weighted imaging sequences in neurovascular compression. World Neurosurg.

[13] Tian X, Tian B, Shi Z, Wu X, Peng W, Zhang X, Malhotra A, Mossa-Basha M, Sekhar L, Liu Q, Lu J, Hu C, Zhu C (2021). Assessment of intracranial atherosclerotic plaques using 3D black-blood MRI: comparison with 3D time-of-flight MRA and DSA. J Magn Reson Imaging.

[14] Engelbrecht MR, Saeed M, Wendland MF, Canet E, Oksendal AN, Higgins CB (1998). Contrast-enhanced 3D-TOF MRA of peripheral vessels: intravascular versus extracellular MR contrast media. J Magn Reson Imaging.

[15] Shang S, Ye J, Dou W, Luo X, Qu J, Zhu Q, Zhang H, Wu J (2019). Validation of zero TE-MRA in the characterization of cerebrovascular diseases: a feasibility study. AJNR Am J Neuroradiol.

[16] Arai N, Akiyama T, Fujiwara K, Koike K, Takahashi S, Horiguchi T, Jinzaki M, Yoshida K (2020). Silent MRA: arterial spin labeling magnetic resonant angiography with ultra-short time echo assessing cerebral arteriovenous malformation. Neuroradiology.

[17] Greve T, Sollmann N, Hock A, Zimmer C, Kirschke JS (2021). Novel ultrafast spiral head MR angiography compared to standard MR and CT angiography. J Neuroimaging.

[18] Weiger M, Brunner DO, Dietrich BE, Muller CF, Pruessmann KP (2013). ZTE imaging in humans. Magn Reson Med.

[19] Wu H, Block WF, Turski PA, Mistretta CA, Johnson KM (2013). Noncontrast-enhanced three-dimensional (3D) intracranial MR angiography using pseudocontinuous arterial spin labeling and accelerated 3D radial acquisition. Magn Reson Med.

[20] Irie R, Suzuki M, Yamamoto M, Takano N, Suga Y, Hori M, Kamagata K, Takayama M, Yoshida M, Sato S, Hamasaki N, Oishi H, Aoki S (2015). Assessing blood flow in an intracranial stent: a feasibility study of MR angiography using a silent scan after stent-assisted coil embolization for anterior circulation aneurysms. AJNR Am J Neuroradiol.

[21] Tomura N, Saginoya T, Kokubun M, Horiuchi K, Watanabe Z (2019). Comparison of time-of-flight-magnetic resonance angiography from silent scan magnetic resonance angiography in depiction of arteriovenous malformation of the brain. J Comput Assist Tomogr.

[22] Katsuki M, Narita N, Ishida N, Sugawara K, Watanabe O, Ozaki D, Sato Y, Kato Y, Jia W, Tominaga T (2021). Usefulness of 3 tesla ultrashort echo time magnetic resonance angiography (UTE-MRA, SILENT-MRA) for evaluation of the mother vessel after cerebral aneurysm clipping: case series of 19 patients. Neurol Med Chir.

[23] Fujiwara Y, Muranaka Y (2017). Improvement in visualization of carotid artery uniformity using silent magnetic resonance angiography. Radiol Phys Technol.

